# Supplementary material for: Mechanisms governing the pioneering and redistribution capabilities of the non-classical pioneer PU.1
Source: Nat Commun. 2020 Jan 21;11:402. doi: 10.1038/s41467-019-13960-2 (PMC6972792; doi:10.1038/s41467-019-13960-2)
Supplement: Supplementary file 7 — Source data [file 41467_2019_13960_MOESM7_ESM.zip › Source_Data/FigureS2/FigureS2B_MotifScanOutput/KmeansCluster12/homerResults/motif2.similar.html]

motif2

## Information for motif2

G
C
A
T
C
G
T
A
T
C
G
A
A
T
G
C
G
A
T
C
C
T
G
A
G
T
A
C
G
C
T
A
  
Reverse Opposite:  

C
G
A
T
C
A
T
G
G
A
C
T
C
T
A
G
T
A
C
G
A
G
C
T
G
C
A
T
C
G
T
A
  

|  |  |
| --- | --- |
| p-value: | 1e-122 |
| log p-value: | -2.811e+02 |
| Information Content per bp: | 1.644 |
| Number of Target Sequences with motif | 623.0 |
| Percentage of Target Sequences with motif | 34.67% |
| Number of Background Sequences with motif | 5753.7 |
| Percentage of Background Sequences with motif | 12.50% |
| Average Position of motif in Targets | 100.8 +/- 52.8bp |
| Average Position of motif in Background | 98.6 +/- 65.5bp |
| Strand Bias (log2 ratio + to - strand density) | -0.1 |
| Multiplicity (# of sites on avg that occur together) | 1.17 |
| Motif File: | file (matrix) reverse opposite |

### Similar de novo motifs found

|  |  |  |  |  |  |  |  |
| --- | --- | --- | --- | --- | --- | --- | --- |
| Rank | Match Score | Redundant Motif | P-value | log P-value | % of Targets | % of Background | Motif file |
| 1 | 0.959 | G C A T G C A T C G T A C T G A A G T C A T G C C T G A T G A C G T C A | 1e-120 | -276.882810 | 29.38% | 9.32% | motif file (matrix) |
| 2 | 0.977 | T A G C A C T G G C A T C G T A C T G A G A T C G T A C C T G A G A T C G C T A | 1e-116 | -268.476557 | 25.21% | 7.13% | motif file (matrix) |
| 3 | 0.973 | C A G T A C T G G A C T A C T G A C T G A G C T C G A T C A G T G A T C A G C T C A G T | 1e-110 | -254.856710 | 20.98% | 5.19% | motif file (matrix) |
| 4 | 0.968 | C G A T A C T G C G A T A C T G A C T G A C G T A C G T C G A T G A T C A C G T G C T A A G C T | 1e-95 | -220.993795 | 14.02% | 2.57% | motif file (matrix) |
| 5 | 0.968 | A C G T A C T G A G C T A C T G A C T G A C G T A C G T | 1e-93 | -215.140646 | 16.42% | 3.71% | motif file (matrix) |
| 6 | 0.961 | G C A T G T C A C G T A T C G A A G T C A G T C C G T A A G T C C G T A T C A G T C A G T C A G G C T A | 1e-78 | -181.257227 | 11.80% | 2.21% | motif file (matrix) |
| 7 | 0.912 | T G C A G T C A T G C A T C G A G A T C A G T C G C T A G T A C G C T A C A T G A T G C G T C A G T C A G C T A | 1e-77 | -178.320276 | 18.81% | 5.69% | motif file (matrix) |
| 8 | 0.841 | G C A T C G T A T C G A A G T C A G T C C T A G A T G C | 1e-73 | -168.384341 | 47.36% | 26.82% | motif file (matrix) |
| 9 | 0.934 | C T G A T G C A A G T C A T G C G C T A T A G C C T G A | 1e-67 | -155.174154 | 54.70% | 34.18% | motif file (matrix) |
| 10 | 0.810 | A G C T A T C G A C G T C T A G A C T G A G T C G C A T C T A G T A C G | 1e-62 | -143.370152 | 37.67% | 20.14% | motif file (matrix) |
| 11 | 0.775 | G T A C A C G T A C T G A C G T C T A G T C A G A G T C G T A C C G A T T A C G T G C A | 1e-55 | -128.432064 | 48.30% | 30.09% | motif file (matrix) |
| 12 | 0.794 | C T A G T C A G C T G A C T A G T C A G T G C A C T G A T C G A T G A C A G T C C T G A T A G C G T C A | 1e-49 | -113.745088 | 8.40% | 1.79% | motif file (matrix) |
| 13 | 0.796 | T G A C C G A T C T A G A G C T A C T G C T A G G A C T G A C T A C G T A G T C A G T C G A C T | 1e-34 | -78.749577 | 4.12% | 0.58% | motif file (matrix) |
| 14 | 0.787 | C T A G G T A C C G T A T A G C G C T A A T C G C G A T A T C G G A C T A C T G C T A G A C G T A C G T C A G T | 1e-17 | -40.153939 | 2.06% | 0.29% | motif file (matrix) |
